# Young ERC Masterclass journal club article: the TOMAHAWK trial

**DOI:** 10.1016/j.resplu.2025.101015

**Published:** 2025-06-25

**Authors:** Alanowd Alghaith, Jan Pupkes, Kristin Alm-Kruse, Godwin Awuni Anafo, Enrico Baldi

**Affiliations:** a Department of Emergency Medical Services, College of Applied Medical Science, King Saud bin Abdulaziz University for Health Science, Riyadh, Saudi Arabia; b King Abdullah International Medical Research Center, Riyadh, Saudi Arabia; c Social Science Applied Healthcare and Improvement Research (SAPPHIRE) Group, Department of Population Health Sciences, College of Life Sciences, University of Leicester, Leicester, United Kingdom; d Tübingen Institute for Medical Education (TIME), Eberhard Karls University, Tübingen, Germany; e Department of Research and Quality Improvement, Division of Prehospital Services, Oslo University Hospital, Oslo, Norway; f Department of Anesthesia and Intensive Care, IRCCS San Raffaele Scientific Institute, Milan, Italy; g Korle-Bu Teaching Hospital, Korle-Bu, Accra, Ghana; h Division of Cardiology, Fondazione IRCCS Policlinico San Matteo, Pavia, Italy; i Cardiac Arrest and Resuscitation Science Research Team (RESTART), Fondazione IRCCS Policlinico San Matteo, Pavia, Italy

## Abstract

### Graphical illustration/graphical abstract

This journal club article is part of the 2023/24 Young ERC Masterclass.^1^ It discusses the paper “Angiography after Out-of-Hospital Cardiac Arrest without ST-Segment Elevation”^2^ from the TOMAHAWK-trial (Fig. 1).

**Fig. 1:**
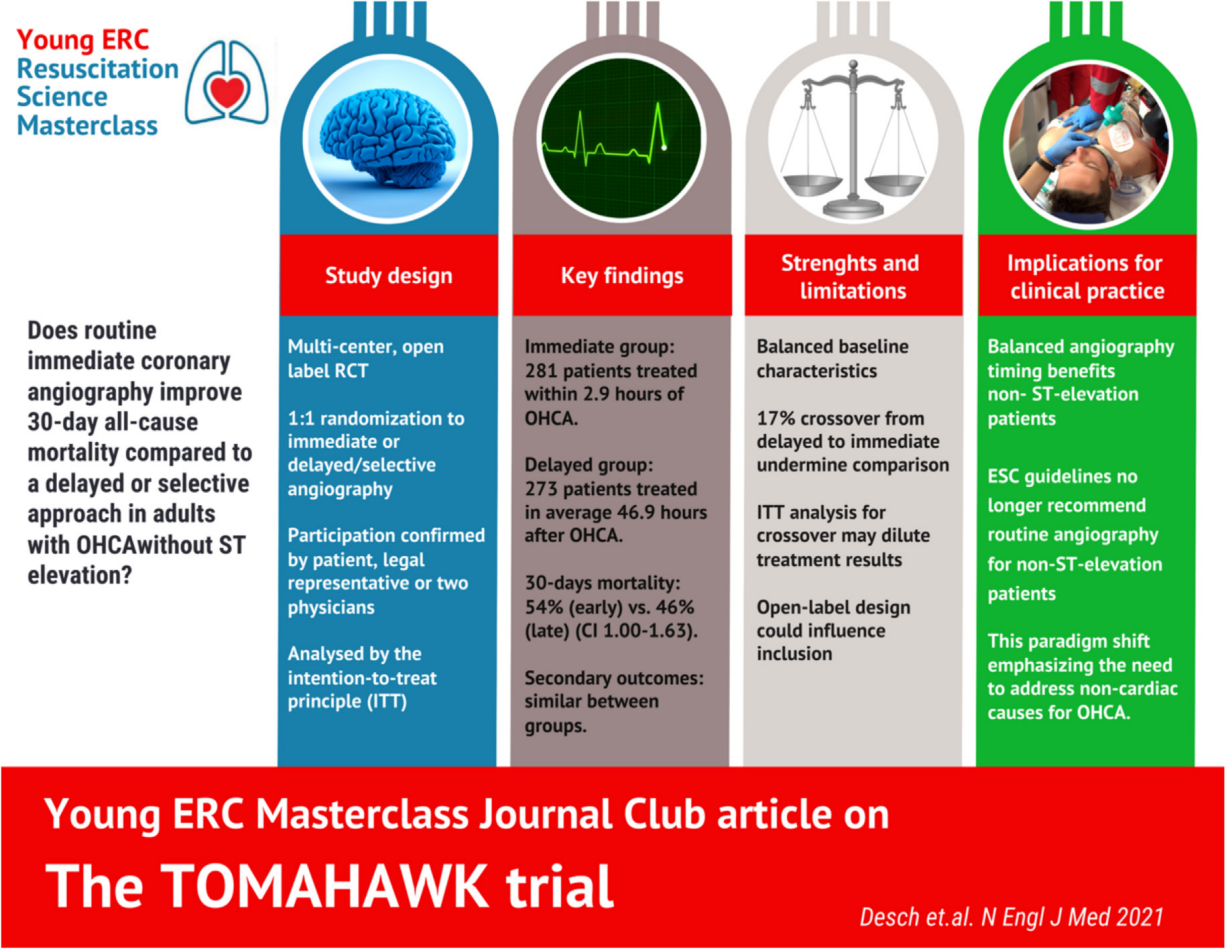
Graphical abstract (TOMAHAWK-trial).

## Which knowledge gap does this study try to fill out?

The Immediate Unselected Coronary Angiography versus Delayed Triage in Survivors of Out-of-Hospital Cardiac Arrest without ST-segment Elevation (TOMAHAWK) trial addresses the knowledge gap in managing Out-Of-Hospital Cardiac Arrest (OHCA) patients without electrocardiographic (ECG) ST-segment elevation after Return of Spontaneous Circulation (ROSC).^2^ While the European Society of Cardiology (ESC) recommends early angiography for Acute Myocardial Infarction (AMI) with ST elevation, the role of this intervention in OHCA patients without ST elevation^3^ remains unclear. Previous studies, such as the Coronary Angiography after Cardiac Arrest without ST-Segment Elevation (COACT) trial,^4^ primarily focused on patients with shockable rhythms. In contrast, the TOMAHAWK trial aimed to determine if immediate angiography improves 30-day mortality compared to a delayed or selective approach in both shockable and non-shockable rhythms.

## What was the design of the study?

The TOMAHAWK trial was a multi-center, open label, Randomized Controlled Trial (RCT). It included adults aged 30 years and older who were successfully resuscitated after OHCA without ST-segment elevation at post-ROSC ECG. Participants were randomized in a 1:1 ratio to immediate or delayed/selective coronary angiography. A power calculation estimated that 558 patients were needed to achieve 80 % power. Safety aspects were overseen by a data and safety monitoring board. In the acute stage, trial participation was confirmed by the patient or a legal representative, if possible, or by two physicians. In the subacute stage, consent had to be obtained from the legal representative or the patient upon regaining acceptable neurological status. All patients were analyzed according to the Intention-To-Treat (ITT) principle.

## What were the key findings of the study?

The TOMAHAWK trial randomized 554 patients: 281 to immediate angiography and 273 to delayed angiography. The immediate group underwent coronary angiography within 2.9 h of cardiac arrest, while the delayed group underwent coronary angiography after an average of 46.9 h. Distribution of shockable and non-shockable rhythms was similar between groups. Physicians selected Percutaneous Coronary Intervention (PCI) or Coronary Artery Bypass Grafting (CABG) based on patient need, and the control group received intensive care before delayed angiography.

At 30 days, the mortality rate was 54 % in the immediate angiography group compared to 46 % in the delayed/selective angiography group (HR: 1.28; 95 % CI: 1.00 to 1.63). This does not suggest a clear benefit of early angiography because the confidence interval includes 1.0, indicating the true effect could range from no difference to a 63 % higher risk of death with immediate angiography. Therefore, it is uncertain whether immediate angiography is harmful or has no effect on outcomes.

Secondary outcomes were similar between groups, including the risk of myocardial infarction (0 % vs. 0.8 %), rehospitalization for heart failure (0.4 % vs. 0.4 %), Intensive Care Unit (ICU) stay (7 vs. 8 days), and moderate/severe bleeding (RR: 1.34; 95 % CI: 0.57 to 3.14). However, the immediate angiography group had a higher incidence of 30-day mortality or major neurological deficit (64.3 % vs. 55.6 %).

Most deaths were due to major neurological injury, and two-thirds of patients had cardiac arrest unrelated to coronary artery disease. Contributing factors may include non-shockable rhythms, coronary artery disease, delayed ROSC, and lack of bystander Cardiopulmonary Resuscitation (CPR).

## Are there any important methodological considerations to learn from the study?

A key methodological theme in this study is trial integrity in the face of treatment crossovers and potential bias. The TOMAHAWK trial had a significant crossover rate (17 %) in the delayed angiography group, with patients undergoing angiography within 24 h despite being randomized to a delayed strategy, which introduces bias. Those who crossed over were likely to have benefited from early angiography, potentially diluting the treatment effect. Such noncompliance complicates interpretation and threatens the internal validity of the trial. While deviations from the protocol may improve patient care, they risk undermining clear comparisons between groups. In clinical trials, crossovers can be minimized by employing clear eligibility criteria, standardized decision algorithms, strict protocol adherence, and robust training of site investigators, while allowing prespecified, protocol-driven criteria for early intervention. However, in emergency settings like OHCA, ethical considerations often necessitate flexibility in treatment decisions, making it challenging to prevent crossover entirely.

In analysis, approaches such as ITT remain primary, but supplementary analyses (per-protocol, as-treated, instrumental variable methods, or complier average causal effect analyses) can help assess the impact of noncompliance. Notably, TOMAHAWK’s ITT and sensitivity analyses yielded consistent results (HR ∼1.28), suggesting crossover did not alter conclusions. Transparent reporting of crossover reasons and handling in both design and analysis phases also enhances interpretability.

The open-label design allowed real-time decisions by clinicians, which is important in critical care settings. However, this approach is vulnerable to performance bias because knowledge of patient allocation can influence decisions and lead to crossovers, as seen in this study. This makes it difficult to isolate the effects of the intervention. The relevance of the performance bias, however, depends on the endpoint. In TOMAHAWK, the primary endpoint was 30-day all-cause mortality, an objective outcome less susceptible to subjective influence. However, open-label status could indirectly affect mortality through clinician behavior (e.g. differential use of adjunctive therapies), though this is less likely than bias in subjective outcomes (e.g., quality of life). To mitigate potential bias in open-label acute trials, standardized care pathways and blinded judgment of outcomes are important.

A double-blinded RCT could address these issues by minimizing bias, especially performance bias.

Blinding in interventional studies is challenging but can be achieved with sham procedures, like sham PCI, where angiography is done without therapeutic intervention. This prevents patients and clinicians from knowing the treatment allocation, reducing bias. Despite ethical concerns, sham procedures can enhance trial integrity.^5^

## What are the most important strengths and limitations of the study?

The study showed robustness with balanced baseline characteristics across treatment groups, with differences due to the interventions. Including patients with different heart rhythms increased generalizability. The TOMAHAWK study had a high follow-up rate (95.7 %). These results align with prior studies, like the COACT trial, indicating early angiography may not be advantageous for this patient group, thereby increasing confidence in findings.^4^ However, there were constraints: 17 % of patients in the delayed/selective angiography group underwent coronary angiography within 24 h, and 4 % of patients were not included in the primary outcome analysis, reducing statistical power from 80 % to 79 %. The power calculation was based on an overly optimistic expectation of a 12 % difference in 30-day mortality. Beyond crossover and power limitations, initial recruitment rates were lower than anticipated, raising the possibility of selection bias, as patients enrolled may differ systematically from those not recruited. Also, protocol changes regarding exclusion criteria for haemodynamic instability or cardiogenic shock occurred, which could affect generalizability and introduce bias if criteria shifted during enrollment. Open-label design and limited longer-term outcomes further constrain interpretation. Transparent discussion of these elements will provide a balanced appraisal.

## How will the results affect your clinical practice?

Along with other recent RCTs, the TOMAHAWK trial has informed the International Liaison Committee on Resuscitation Consensus on Science with Treatment Recommendations  (ILCOR CoSTR) of 2022,^6^ emphasizing the lack of benefit of immediate angiography in OHCA patients without ST-segment elevation. These findings support a nuanced approach to post-resuscitation care in guidelines, balancing the timing of coronary angiography with addressing potential non-coronary causes of cardiac arrest. The 2023 ESC Guidelines for Acute Coronary Syndromes^3^ have already incorporated these findings, stating that routine immediate angiography after resuscitated CA is not recommended in hemodynamically stable patients without ST-segment elevation. However, the current European Resuscitation Council  (ERC) 2021 guidelines^7^ still state that it should be considered, making the CoSTR results particularly relevant for future guideline discussions. With the upcoming 2025 ERC Guideline update, this evidence may contribute to a paradigm shift, potentially aligning ERC recommendations more closely with ESC. This represents a paradigm shift for healthcare professionals, highlighting the need to rule out non-coronary causes (e.g. cerebrovascular events, respiratory failure, non-cardiogenic shock, pulmonary embolism, or intoxication) before coronary angiography.

## What do you see as the next steps in research?

Looking at the 2023 ESC guidelines for the management of acute coronary syndromes, it is evident how ST-segment elevation on the post-ROSC ECG plays a major role in determining the patient’s treatment. Absence of ST-segment elevation leads to a class III recommendation for immediate coronary angiography, while the presence of ST-segment elevation leads to a class I recommendation for immediate coronary angiography. However, a recent meta-analysis indicates low sensitivity of ST-segment elevation for acute coronary lesion and revascularization at the first ECG after ROSC.^8^ This is likely due to differing diagnostic criteria and timing of ECG acquisition among studies. Too early ECG acquisition post-ROSC is associated with more false-positive ST-Elevation Myocardial Infarction (STEMI) cases,^9^ possibly due to absence of coronary flow during CA causing transmural myocardial ischemia of non-coronary origin. Considering the recent ESC Guidelines and the need for accurate STEMI diagnosis in managing post-ROSC patients, large prospective studies are necessary to determine optimal timing for ECG recording post-ROSC to review ECG criteria for post-ROSC STEMI, and evaluate risk–benefit for borderline cases. Ongoing research, such as the DISCO trial,^10^ may also provide further clarity on optimal management strategies for this patient population and future research could assess the real-world implementation of selective angiography strategies and their impact on outcomes.

## Ethical considerations

Not applicable.

## CRediT authorship contribution statement

**Alanowd Alghaith:** Writing – review & editing, Writing – original draft, Visualization, Conceptualization. **Jan Pupkes:** Writing – review & editing, Writing – original draft, Visualization, Conceptualization. **Kristin Alm-Kruse:** Writing – review & editing, Writing – original draft, Visualization, Conceptualization. **Godwin Awuni Anafo:** Writing – review & editing, Writing – original draft, Visualization, Conceptualization. **Enrico Baldi:** Writing – review & editing, Writing – original draft, Visualization, Supervision, Conceptualization.

## Declaration of competing interest

The authors declare that they have no known competing financial interests or personal relationships that could have appeared to influence the work reported in this paper.
